# *Pax9*’s Interaction With the Ectodysplasin Signaling Pathway During the Patterning of Dentition

**DOI:** 10.3389/fphys.2020.581843

**Published:** 2020-11-26

**Authors:** Shihai Jia, Jeremie D. Oliver, Emma C. Turner, Maranda Renouard, Marianna Bei, J. T. Wright, Rena N. D’Souza

**Affiliations:** ^1^School of Dentistry, University of Utah Health, Salt Lake City, UT, United States; ^2^Department of Biomedical Engineering, College of Engineering, The University of Utah, Salt Lake City, UT, United States; ^3^Dental School, Faculty of Health and Medical Sciences, The University of Western Australia, Perth, WA, Australia; ^4^College of Pharmacy, University of Utah Health, Salt Lake City, UT, United States; ^5^Massachusetts General Hospital, Harvard Medical School, Boston, MA, United States; ^6^Adams School of Dentistry, The University of North Carolina at Chapel Hill, Chapel Hill, NC, United States; ^7^Department of Neurobiology and Anatomy, Pathology, and Surgery, The University of Utah, Salt Lake City, UT, United States

**Keywords:** tooth development, signaling interaction, patterning, incisor development, *Pax9*, ectodysplasin pathway

## Abstract

In these studies, we explored for the first time the molecular relationship between the paired-domain-containing transcription factor, *Pax9*, and the ectodysplasin (*Eda*) signaling pathway during mouse incisor formation. Mice that were deficient in both *Pax9* and *Eda* were generated, and the status of dentition analyzed in all progeny using gross evaluation and histomorphometric means. When compared to wildtype controls, *Pax9^+/–^Eda^–/–^* mice lack mandibular incisors. Interestingly, *Fgf* and *Shh* signaling are down-regulated while *Bmp4* and *Lef1* appear unaffected. These findings suggest that *Pax9*-dependent signaling involves the *Eda* pathway and that this genetic relationship is important for mandibular incisor development. Studies of records of humans affected by mutations in *PAX9* lead to the congenital absence of posterior dentition but interestingly involve agenesis of mandibular central incisors. The latter phenotype is exhibited by individuals with *EDA* or *EDAR* mutations. Thus, it is likely that *PAX9*, in addition to playing a role in the formation of more complex dentition, is also involved with *EDA* signaling in the initiation of odontogenesis within the incisal domain.

## Introduction

The formation of mammalian dentition is a remarkable developmental process and a valuable model for studying epithelial–mesenchymal signaling interactions that control patterning morphogenesis. Much of our understanding about the patterning of dentition comes from mouse studies. The use of transgenesis, gene targeting, expression analyses, functional tooth recombination, as well as bead implantation assays have advanced our knowledge about the patterning of the murine dentition. What has emerged is the realization that tooth development involves a complex series of genetic interactions between growth factors, transcription factors, signal receptors, and diffusible morphogens that interact within five critical pathways, namely, bone morphogenetic protein (Bmp), wingless-integrated site (Wnt), fibroblast growth factor (Fgf), sonic hedgehog (Shh), and ectodysplasin (Eda) (Lan et al., 2014; Balic and Thesleff, 2015; Huang et al., 2019).

That the patterning of dentition is under strict genetic control is best underscored by the condition of human tooth agenesis, a common inherited disorder that affects over 20% of the population. Classified as genetically and phenotypically heterogeneous, tooth agenesis most commonly affects third molars, mandibular second premolars, maxillary lateral incisors, and maxillary second premolars (Kapadia et al., 2007; Nieminen, 2009; Ye and Attaie, 2016; Williams and Letra, 2018). These commonly missing teeth represent the most distal members of each tooth family and fail to develop due to a disruption in normal signaling. This suggests that distinct distal-proximal morphogenetic gradients are involved in guiding the patterning of human dentition.

The appearance of tooth placodes marks the onset of odontogenesis and the formation of incisiform and molariform fields that develop when the inductive potential from dental epithelium is transferred to underlying mesenchyme (Jarvinen et al., 2018; Balic, 2019). This was proven in elegant recombination experiments where bud-staged molar mesenchyme was shown to fully transform non-dental epithelium to form teeth (Mina and Kollar, 1987; Lumsden, 1988). Systems biology approaches that integrated data from genome-wide expression profiling, bioinformatics, and *in vivo* genetic models show that canonical Wnt signals are the primary drivers of tooth signaling interactions (O’Connell et al., 2012). *Lef1*, a nuclear mediator of Wnt signaling, associates with β-catenin and activates Wnt-responsive genes. The molecular relationship between Wnt and Eda signaling pathways is well established in tooth development as *Eda* is down-regulated in *Lef1^–/–^* tooth organs that are arrested at the bud stage and Wnt6 can induce *Eda* expression in a *Lef1*-dependent manner (Laurikkala et al., 2001). Multiple lines of evidence further converge to support the hypothesis that the interactions between signaling pathways, rather than the intrinsic functions of transcription factors alone, dictate how the patterning of dentition is orchestrated.

Several studies point to the importance of the homeodomain-containing transcription factor, *Pax9*, as a key mediator of the odontogenic potential in the mesenchyme (Neubuser et al., 1997; Peters et al., 1998; Jernvall and Thesleff, 2000; Kapadia et al., 2006; Ogawa et al., 2006; Chen et al., 2009). Although *Pax9*-dependent signaling in tooth mesenchyme involves a partnership with *Msx1* and the up-regulation of *Bmp4* expression, its relationship with other key pathways is not well understood. These studies explored for the first time the relationship of *Pax9* with the Eda signaling pathway during the formation of mouse dentition. Through the use of mouse genetics, we demonstrate that *Pax9*-dependent signaling is functionally integrated with Eda signaling during mandibular incisor development. Disruptions in this molecular relationship lead to downregulation of other signaling molecules. We also report on the findings of our long-term human genetic studies that show that mutations in *PAX9*, while dominantly affecting posterior dentition, often involve mandibular central incisors. The latter, we observe is a common occurrence in individuals with mutations in *EDA* or *EDAR*. Taken together, our results indicate that *Pax9*-mediated signaling involves the Eda pathway, impacting the initiation of odontogenesis within the mandibular incisiform field.

## Materials and Methods

### Mouse Strains

All animal procedures were approved by the Institutional Animal Care and Use Committee (IACUC) at the University of Utah (Protocol #19-12012). *Pax9*^+/–^ mice were provided by Dr. Rulang Jiang (Cincinnati Children’s Hospital), and *Eda*^+/–^ mice (# 000314) were purchased from Jackson laboratory. Mouse colonies were maintained in the *C57BL/6* background, and 2–8-month-old females were used for intercross mating.

### Histology Analysis

For the whole-mount overview of mandibles and maxillae, 2-month-old mouse heads were removed using fine forceps under a stereomicroscope. Mandibles were carefully dissected after removal of the tongue, and images were taken through a stereomicroscope (Zeiss Stemi 508).

For H&E staining, whole heads were fixed in 4% paraformaldehyde (PFA) in PBS overnight and processed through serial gradients of ethanol and xylene for paraffin embedding. 7 μm-thick sagittal sections were stained with H&E and evaluated under a digital microscope (EVOS).

### *In situ* Hybridizations

Sagittal paraffin sections of E13.5 and E14 embryos using digoxigenin-labeled RNA probes (1 μg/ml) as described previously (Jia et al., 2017). Embryo heads were fixed in 4% PFA in PBS overnight then processed through serial ethanol and xylene for paraffin embedding. 7 μm-thick sagittal sections were hybridized with digoxigenin-labeled antisense RNA probes (1 μg/ml) to *Bmp4*, *Fgf3*, *Lef1*, and *Shh* as described previously. An anti-digoxigenin-AP antibody (11093274910, ROCHE, 1:1000) was used to detect the labeled probe. Comparable images were taken with a digital microscope (EVOS). At least three biological replicates were used to establish the reproducibility of results.

### Tooth Germ Dissections and Real-Time Reverse Transcription (RT)-PCR Analyses

The embryos heads were harvested at E13.5 in cold PBS. After the lower jaw and brain were removed under a dissecting microscope, the incisors were carefully dissected using fine forceps and stored individually at −80°C for total RNA extraction. After genotyping, 3 pairs of incisors were pooled and total RNA was extracted using the RNeasy Micro Kit (Qiagen). First-strand cDNA was synthesized using the SuperScript First-Strand Synthesis System IV (Thermo Fisher Scientific). Quantitative reverse transcription (RT)-PCRs were performed using the SYBR Green^ER^ qPCR Supermix (Thermo Fisher Scientific). A list of gene-specific primers is provided in Supplementary Table S1. For each sample, the relative levels of target mRNAs were normalized to *Gapdh* using the standard curve method (Zhou et al., 2013). Three sets of biological replicates were analyzed for each gene.

### Clinical Findings From Human Genetic Studies

Patients were identified and evaluated after approval was obtained from the Committee for the Protection of Human Subjects, University of Texas Health Science Center at Houston. Consent to participate (including a release of dental records) was obtained from a parental guardian, in the case of minors. Patient records are secured at the University of Utah and analyzed for these studies under HIPAA policies and without any personal identifiers. Medical and dental records of patients diagnosed with mutations in *PAX9* and *EDAR* were studied along with radiographs and photographs. Patterns of tooth agenesis were noted, and records were selected to illustrate mandibular incisor agenesis in two individuals with mutations in *PAX9* and *EDAR*, respectively.

## Results

### *Pax9^+/–^Eda^–/–^* Compound Mutant Mice Exhibited Fewer Molars and Missing Mandibular Incisors

**FIGURE 1:**
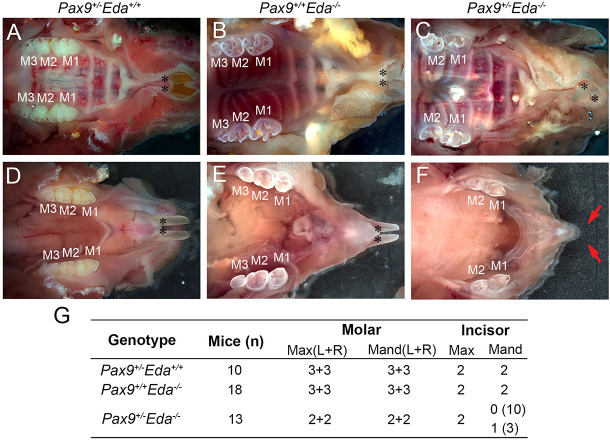
Missing teeth in *Pax9^+/–^Eda^–/–^* compound mutant mice. **(A–C)** Whole-mount view of maxillary teeth in 2-month-old mice. **(A)** *Pax9^+/–^Eda^+/+^* mice showed six molars (3 + 3) and two incisors. **(B)** *Pax9^+/+^Eda^–/–^* mice showed six molars (3 + 3) with fewer cusps and two incisors. **(C)** *Pax9^+/–^Eda^–/–^* mice showed four molars (2 + 2) and two incisors. **(D–F)** Whole-mount view of mandibular teeth in 2-month-old mice. **(D)** *Pax9^+/–^Eda^+/+^* mice showed two incisors and six molars (3 + 3). **(E)** *Pax9^+/+^Eda^–/–^* mice showed two incisors and six molars (3 + 3) with smaller size and fewer cusps. **(F)** *Pax9^+/–^Eda^–/–^* mice showed missing incisors and four molars (2 + 2) with smaller size and fewer cusps. **(G)** Summary of teeth phenotype in *Pax9^+/–^Eda^–/–^* compound mutants. Red arrows pointed to the space of missing incisors. M1, M2, and M3 are first, second, and third molar, respectively. Black * presents incisors. Max, maxillary; Mand, mandible.

*Pax9*^+/–^ and *Eda*^+/–^ mice exhibited normal dentition with no disruptions in the size, shape, and number of teeth. To begin to assess whether *Pax9* and *Eda* genetically interact during tooth development, *Pax9^+/–^Eda^–/–^* mice were generated through a series of breeding. As shown in Figures 1A,D, *Pax9^+/–^Eda^+/+^* mice had a full complement of six molars (3 + 3) and two incisors in each mandible and maxilla, respectively. The 2-month-old *Pax9^+/+^Eda^–/–^* mice (Figures 1B,E) had smaller molars with shallower cusps (as reported earlier) with six molars (3 + 3) and two incisors present in each mandible and maxilla analyzed. The compound mutant *Pax9^+/–^Eda^–/–^* mice exhibited missing third molars in both the mandible and maxilla, as well as missing mandibular incisors (Figures 1C,F,G). These data suggest that *Pax9* and *Eda-*dependent signaling pathway genes share a genetic interaction in controlling the molar number and the formation of mandibular incisors.

### Mandibular Incisor Development in *Pax9^+/–^Eda^–/–^* Compound Mutants

**FIGURE 2:**
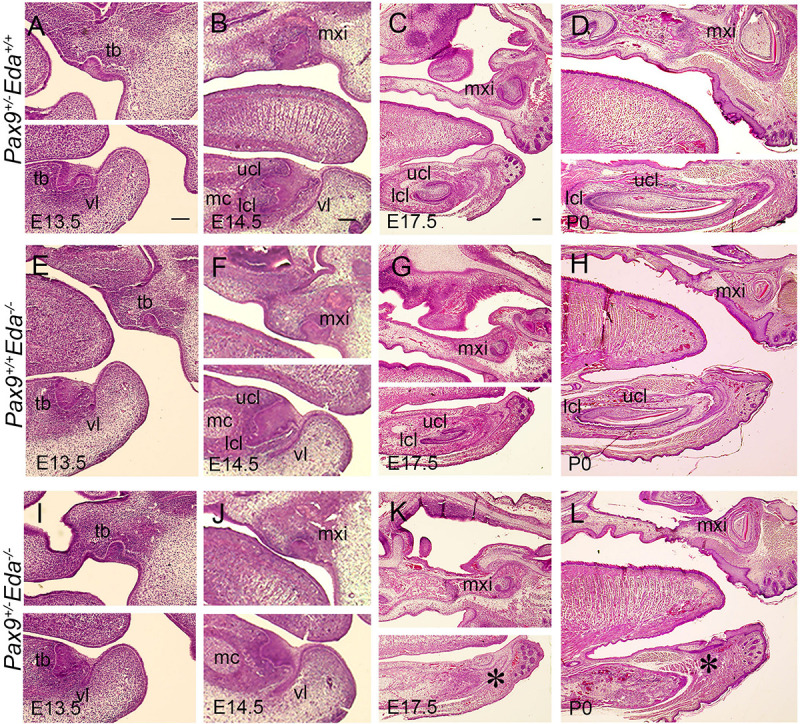
Incisor developmental defects in the *Pax9^+/–^Eda^–/–^* compound mutant embryos. Hematoxylin and eosin-stained (HE) sagittal sections through the developing incisor tooth germs. The *Pax9^+/–^Eda^+/+^*, *Pax9^+/+^Eda^–/–^*, and *Pax9^+/–^Eda^–/–^* compound mutants at E13.5 **(A,E,I)**, E14.5 **(B,F,J)**, E17.5 **(C,G,K)**, and P0 **(D,H,L)**, respectively, are shown. Scale bar presented 100 μm, * indicates the region of missing incisor. lcl, lower cervical loop; mc, Meckel’s cartilage; mxi, maxillary incisor; tb, tooth bud; ucl, upper cervical loop, vl, vestibular lamina.

We examined embryos from E13.5 to P0, and found that at E13.5, the mandibular incisors formed bud-shape tooth germs in *Pax9^+/–^Eda^+/+^, Pax9^+/+^Eda^–/–^*, and *Pax9^+/–^Eda^–/–^* compound mutant mice (Figures 2A,E,I). At E14.5, the cervical loops of *Pax9^+/–^Eda^+/+^* and *Pax9^+/+^Eda^–/–^* mandibular incisors were well formed, whereas the cervical loops of *Pax9^+/–^Eda^–/–^* compound mutant mandibular incisors appeared under-developed (Figures 2B,F,J). At P0, mandibular incisors showed asymmetric cervical loops at E17.5 and well-differentiated tooth organ layers in *Pax9^+/–^Eda^+/+^* and *Pax9^+/+^Eda^–/–^* mandible (Figures 2C,D,G,H). In contrast, the mandibular incisors in *Pax9^+/–^Eda^–/–^* compound mutant exhibited residue of retarded tooth germs (Figures 2K,L).

### The Activity of *Fgf* and *Shh* Signaling Was Reduced in *Pax9^+/–^Eda^–/–^* Mandibular Incisors

**FIGURE 3:**
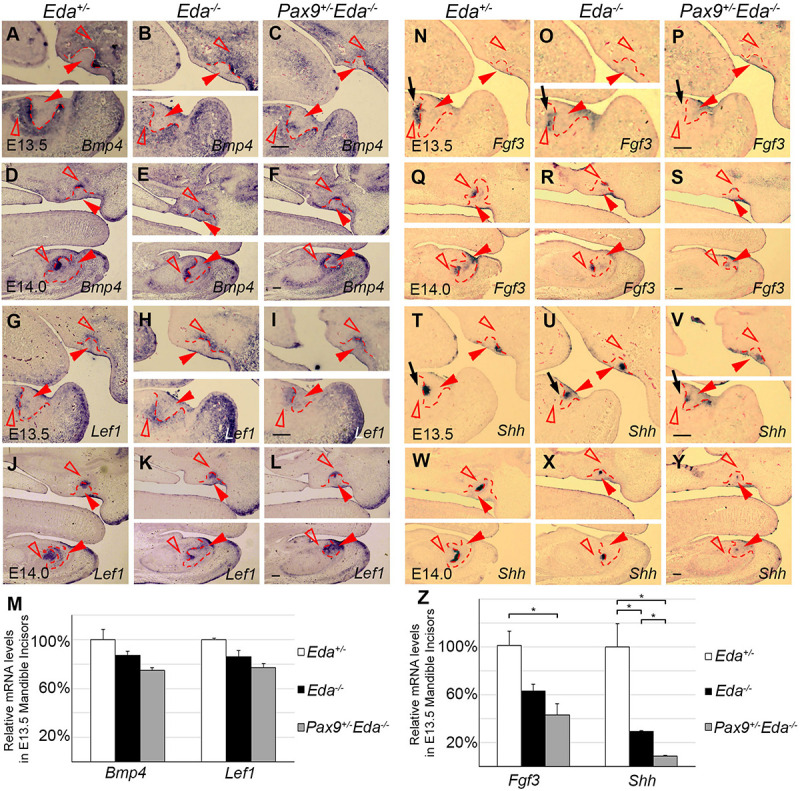
Comparison of incisor molecular marker expression in E13.5 and E14.0 tooth germs. The mRNA expression of *Bmp4* and *Lef1* were shown in *Eda*^+/–^, *Eda^–/–^*, and *Pax9^+/–^Eda^–/–^* mutants by *in situ* hybridization at E13.5 **(A–C,G–H)**, as well as at E14.0 **(D–F,G–L)**. The expression of *Fgf3* and *Shh* was shown in *Eda*^+/–^, *Eda^–/–^*, and *Pax9^+/–^Eda^–/–^* mutants by *in situ* hybridization at E13.5 **(N–P,T–V)**, as well as at E14.0 **(Q–S,W–Y)**. Scale bar presented 100 μm, black arrows indicate mandibular incisor tooth germ. Red solid arrowheads indicate dental epithelia and red open arrowheads indicate dental mesenchyme. Red dashed lines indicate the boundary of incisor tooth germ mesenchyme and epithelia. **(M,Z)** The relative mRNA levels of *Bmp4*, *Lef1*, *Fgf3*, and *Shh* were analyzed by quantitative RT-PCR using the microdissected E13.5 mandibular incisors (*n* = 3). Error bars indicate SEM, *P < 0.01.

To investigate the potential interactions between *Pax9* and the Eda/r signaling pathway, we analyzed the expression patterns of *Bmp4*, *Fgf3*, *Lef1*, and *Shh* in E13.5 and E14.0 *Eda*^+/−^, *Eda*^–/–^, and *Pax9^+/–^Eda^–/–^* incisor organs. Results of *in situ* hybridization indicate that the patterns of *Bmp4* and *Lef1* expression seen in both *Eda*^+/–^ and *Eda^–/–^* incisor organs resembled that visible in *Pax9^+/–^Eda^–/–^* embryos (Figures 3A–L). The expression level of *Bmp4* and *Lef1* was not significantly reduced in the *Pax9^+/–^Eda^–/–^* mandibular incisors confirmed by quantitative RT-PCR (Figure 3M). In contrast, the expression pattern of *Fgf3* and *Shh* appeared down-regulated in *Pax9^+/–^Eda^–/–^* incisors in comparison to that evident in both *Eda*^+/–^ and *Eda^–/–^* samples (Figures 3N–Y). Quantitative RT-PCR revealed that the expression of *Fgf3* in the *Pax9^+/–^Eda^–/–^* mandibular incisor was significantly reduced in comparison to that in the *Eda*^+/–^ samples; and the expression of *Shh* in the *Pax9^+/–^Eda^–/–^* mandibular incisor was significantly reduced compared with that in both *Eda*^+/–^ and *Eda^–/–^* samples (Figure 3Z).

### Patterns of Missing Teeth in Individuals With *PAX9* and *EDAR* Mutations

**FIGURE 4:**
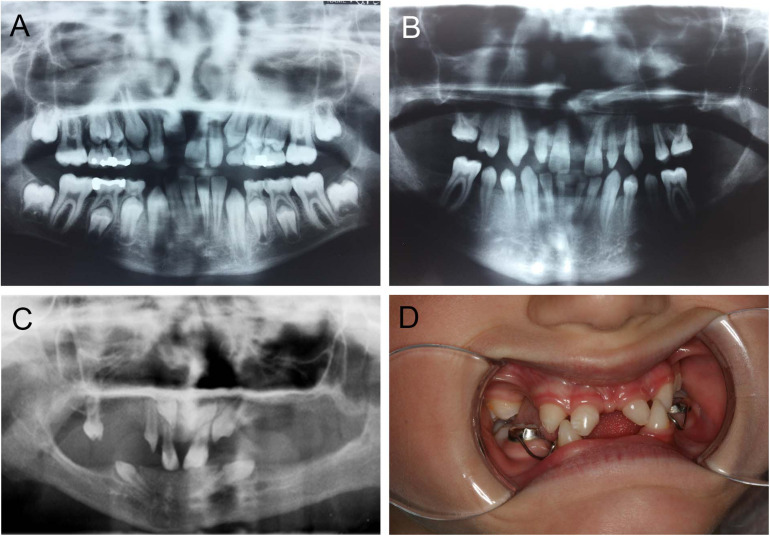
Abnormal tooth appearance, including agenesis of mandibular central incisors, premolars, and molars in a *PAX9* mutant patient **(B)** compared to an unaffected sibling with normal permanent dentition **(A)**; 5-year-old patient with a confirmed mutation of *EDA* gene (XLHED), no permanent teeth in the mandible and only primary canines (never erupted), conical incisors, canines, and one molar noted in the maxilla **(C)**; female patient with autosomal recessive (AR) HED, missing mandibular incisors, and first primary molars, as well as maxillary lateral incisors and first primary molars **(D)**.

Mutations in *PAX9* result in a pattern of tooth agenesis that dominantly involves permanent maxillary and mandibular first, second, and third molars along with second premolars. However, agenesis of mandibular central incisors are often associated with missing molars and premolars, as shown in Figure 4B, and as previously reported by our group (Goldenberg et al., 2000; Stockton et al., 2000; Frazier-Bowers et al., 2002a,b). A sibling with an unaffected *PAX9* gene shows a normal complement of permanent teeth (Figure 4A). For individuals with mutations in *EDA* or *EDAR*, tooth agenesis is more severe and mixed, typically including maxillary and central incisors while frequently involving mandibular central incisors (Figures 4C,D).

## Discussion

The patterning of dentition is a complex and unique developmental process involving multiple genes that control the precise shape, size, number, and position of teeth. While the past decades have advanced our understanding of the transcription factors that are involved, relatively little is known about their interactions with downstream effectors within the Bmp, Fgf, Wnt, Eda, and Shh pathways. Here, we sought to understand the relationship between *Pax9*, a key transcription factor in dental mesenchyme, and the Eda signaling pathway during the development of the dentition. Previous studies indicate that *Pax9* regulates both Wnt and Bmp signaling pathways and Eda signaling has been reported linked with Wnt signaling (Peters et al., 1998; Laurikkala et al., 2001; Zhang et al., 2009; O’Connell et al., 2012; Voutilainen et al., 2015). Our studies show, for the first time, that *Pax9*-dependent signaling is functionally integrated with Eda signaling during odontogenesis. A mouse genetic model deficient in both *Pax9* and *Eda* shows a consistent lack of maxillary and mandibular third molar development. Interestingly, mandibular incisor organs are arrested, a phenotypic change that suggests regionally specific interactions between *Pax9*-dependent signaling and the Eda pathway. In order to fully elucidate the nature of Pax9’s relationship with Eda signaling, further experiments such as chromatin immunoprecipitation (ChIP) or electrophoretic mobility shift assay (EMSA) are needed. Our mouse data align with observations from our human genetic analyses of individuals with mutations in *PAX9* and *EDA/R* genes that show agenesis of mandibular central incisors. Taken together, these results underscore the need for further studies that will elucidate the relationship of *Pax9* with other signaling molecules that direct key epithelial–mesenchymal interactions in tooth development.

As reported earlier, *Eda^–/–^* mice exhibit mild defects in dentition that include fewer and shallower cusps in molars, hypoplasia, and agenesis of maxillary and mandibular third molars in 20% of animals studied. These studies also showed that decreased levels of Eda signaling did not affect incisor development (Pispa et al., 1999; Parveen et al., 2019). Without exception, all of the 13 *Pax9^+/–^Eda^–/–^* mice studied showed maxillary and mandibular third molar agenesis with an overall reduction in size and cuspal morphology of residual first and second molars. *Pax9*^+/–^ mice have a full complement of teeth while *Pax9*^–/^*^–^* mice consistently lack all molars (Kist et al., 2005; Mitsui et al., 2014). Furthermore, permanent molars are the dominant tooth group affected in individuals with mutations in *PAX9* (Goldenberg et al., 2000; Stockton et al., 2000; Frazier-Bowers et al., 2002a). Therefore, it is likely that the combinatorial reduction of *Pax9* and Eda signaling in *Pax9^+/–^Eda^–/–^* mice has an additive effect on maxillary and mandibular molar development.

*Pax9^+/–^Eda^–/–^* mice also exhibited a mandibular incisor phenotype as 10 of 13 embryos studied lacked lower incisors while in three of 13 embryos only a single mandibular incisor was present. In mouse genetic models disruptions in *Pitx2*, *BCL11B* and Wnt, Fgf, Bmp, and Wnt signaling pathway genes result in incisor organ agenesis or hypoplasia (Lin et al., 1999; Lu et al., 1999; Millar et al., 2003; Golonzhka et al., 2009; Yang et al., 2013; Yu et al., 2020). Furthermore, supernumerary incisors arise in mice lacking *Spry2/4*, *Sostdc1*, and *Lrp4* (Ohazama et al., 2008; Munne et al., 2009; Charles et al., 2011). In these models, both maxillary and mandibular incisors were affected as compared to mice where the up-regulation of NF-κB activity and the reduction of *Fam20B* selectively affect mandibular vs. maxillary incisors, respectively (Wang et al., 2013; Tian et al., 2015). Our quantitative RT-PCR and *in situ* hybridization data revealed that the expression of *Fgf3* in the *Pax9^+/–^Eda^–/–^* mandibular incisor was significantly reduced in comparison to that in the *Eda*^+/–^ samples. Whereas, in comparison to the *Eda*^+/–^ samples, the expression of *Fgf3* was reduced in the *Eda^–/–^* incisors, but we found *p* > 0.05 with statistic analysis (Figure 3Z). Previous studies showed that *Fgf3*, *Fgf10*, and *Fgf20* were related to tooth development, both *Fgf3* and *Fgf10* were down-regulated in *Pax9^+/–^Msx1^+/–^* embryos with missing mandibular incisors; and treatment with Fgf10 partially rescued the cusp defect in *Eda^–/–^* mouse (Pispa et al., 1999; Nakatomi et al., 2010). The expression of *Fgf20* was reduced in the developing *Eda^–/–^* incisors, while the *Fgf20* null mice had normal incisors (Häärä et al., 2012). Taken together, the Fgf signaling activity was reduced in *Eda^–/–^* tooth germs but appear adequate for regulating tooth formation; in *Pax9^+/–^Eda^–/–^* mandibles, the level of Fgf signaling was reduced to a certain level that appears inadequate for the normal development of mandibular incisors. Furthermore, the expression of *Shh* in the *Pax9^+/–^Eda^–/–^* mandibular incisor was significantly reduced compared with that in both *Eda*^+/–^ and *Eda^–/–^* samples (Figure 3Z). The marker gene analysis indicates that both Fgf and Shh signaling pathways were involved in the genetic interaction between *Pax9* and *Eda* during mandibular incisor formation. These data when viewed in the light of our findings suggest that a distinct cadre of genetic interactions are involved in the formation of mandibular incisors, whose initiation signifies the earliest zone of odontogenic induction in the mandibular arch.

As described by Nakatomi et al. (2010), *Pax9* and *Msx1* double heterozygous mutant mice show a consistent lack of mandibular incisors, a phenotype that is not evident in single mutant strains. Arrested *Pax9^+/–^Msx1^+/–^* mandibular incisor organs show a marked reduction in *Fgf3* and *Fgf10* expression within dental mesenchyme as well as reduced expression of *Shh* and *Bmp2* in the dental epithelium. Our earlier biochemical analyses had first demonstrated that *Pax9* interacts synergistically with *Msx1* (Ogawa et al., 2006), so it is likely that this partnership plays a key role in driving key signaling interactions between dental mesenchyme and epithelium during the transition from the bud to the cap stage of development. Whether Eda signaling pathway genes are down-regulated in *Pax9^+/–^Msx1^+/–^* mandibular incisor organs offers a valuable direction for future studies. Our findings that signaling pathway genes are differentially affected in *Pax9^+/–^Eda^–/–^* mandibular incisor organs also warrant further investigation. In *Pax9^+/–^Eda^–/–^* maxillary incisors, *Shh* expression was slightly changed suggesting that the level of *Shh* was enough to induce the formation of maxillary incisors. Since the expression of *Bmp4* remained unaffected in dental mesenchyme of*Pax9^+/–^Eda^–/–^* compound mandibular incisors, it is possible that the haploinsufficiency of *Pax9* did not compromise *Bmp4* expression in the developing incisor mesenchyme. Although it was reported that Wnt signaling pathway mediator *Lef1* was down-regulated in the skin of *Tabby* mice (Durmowicz et al., 2002), our data showed that the expression of *Lef1* was not affected in *Pax9^+/–^Eda^–/–^* compound mutant, which matched the regulatory hierarchy that *Eda* was downstream of *Lef1* in the tooth organ (Laurikkala et al., 2001).

Patterns of tooth agenesis in humans provide valuable clues about the important roles that transcription factors in modulating epithelial–mesenchymal signaling during tooth development. As one of the best-studied genes, *PAX9*, is largely viewed as important for the patterning of human dentition. Our group’s clinical observations and that of others have documented that mutations in *PAX9* consistently result in a pattern of tooth agenesis that involves posterior dentition, namely, molars and premolars. Intriguing is the finding that individuals with mutations in *PAX9* often miss mandibular central incisors, the least patterned tooth in human dentition. While patients affected with mutations in *EDA* or *EDAR* lack posterior and anterior teeth in both arches, mandibular incisors are most often missing, a pattern of tooth agenesis that resembles that seen in individuals with mutations in *WNT10A* (Mues et al., 2014). Taken together, these data suggest that each tooth family (incisiform and cuspform/molariform) arises from distinct morphogenetic gradients or fields created by the differential actions of transcription factors and signaling pathways. However, the initiation of odontogenesis within each field is spatially regulated by unique molecular relationships such as for mandibular incisors where Pax9-dependent signaling and the Eda pathway appear to play an important role.

## Data Availability Statement

The original contributions presented in the study are included in the article/Supplementary Material. Further inquiries can be directed to the corresponding author.

## Ethics Statement

The studies involving human participants were reviewed and approved by the Committee for the Protection of Human Subjects, University of Texas Health Science Center at Houston. Written informed consent to participate in this study was provided by the participants’ legal guardian/next of kin. The animal study was reviewed and approved by the Institutional Animal Care and Use Committee (IACUC) at the University of Utah (Protocol #19-12012). Written informed consent was obtained from the individual(s), and minor(s)’ legal guardian/next of kin, for the publication of any potentially identifiable images or data included in this article.

## Author Contributions

SHJ and RD’S contributed to the research design, data acquisition, and analyses, as well as the writing of the manuscript. JO contributed to the writing of the manuscript. ET and MR contributed to the mouse genetic data acquisition. MB contributed to the mouse genetic data analysis. JW contributed to the data acquisition for the medical and dental records of the patient. Each author gave final approval and agreed to be accountable for all aspects of the work, ensuring integrity and accuracy. All authors contributed to the article and approved the submitted version.

## Conflict of Interest

The authors declare that the research was conducted in the absence of any commercial or financial relationships that could be construed as a potential conflict of interest.
