# Supplementary material for: Pax9’s Interaction With the Ectodysplasin Signaling Pathway During the Patterning of Dentition
Source: Front Physiol. 2020 Nov 26;11:581843. doi: 10.3389/fphys.2020.581843 (PMC7732595; doi:10.3389/fphys.2020.581843)
Supplement: Supplementary file 1 [file Table_1.docx]

**Supplementary Table 1:**

Primers for Quantitative RT-PCR (ordered from Integrated DNA Technologies)

| **Name** | **Sequence 5’ - 3’** |
| --- | --- |
| *Bmp4-QF* | GAGGGATCTTTACCGGCTCC |
| *Bmp4-QR* | GTTGAAGAGGAAACGAAAAGCAG |
| *Lef1-QF* | GAAATCATCCCAGCCAGCAA |
| *Lef1-QR* | GGGCATCATTATGTAGCCAGAGTA |
| *Shh-QF* | GAATCCAAAGCTCACATCCAC |
| *Shh-QR* | CGTAAGTCCTTCACCAGCTTG |
| *Fgf3-QF* | GAACAGCGCCTATAGCATCC |
| *Fgf3-QR* | TCCACAAACTCACACTCTGC |
| *Gapdh-F* | TGGAGCCAAAAGGGTCA |
| *Gapdh-R* | CTTCTGGGTGGCAGTGA |
